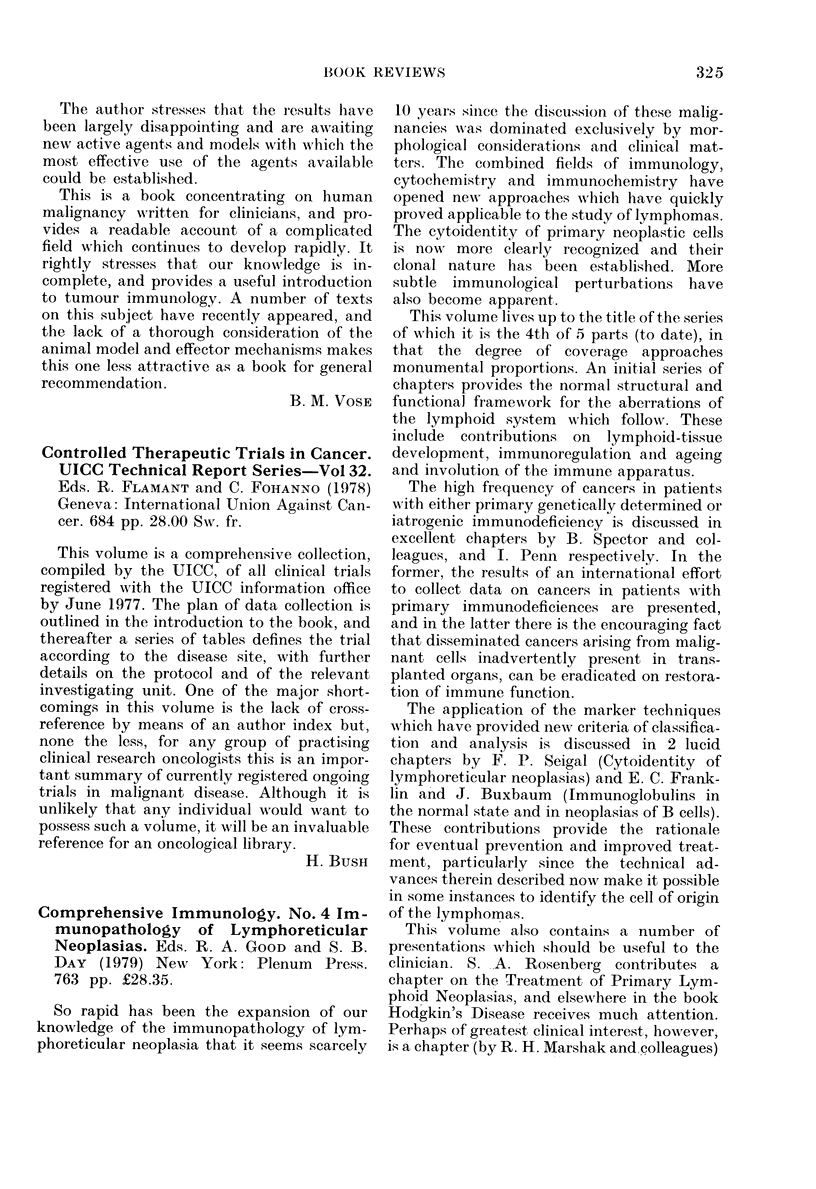# Controlled Therapeutic Trials in Cancer. UICC Technical Report Series—Vol 32

**Published:** 1979-08

**Authors:** H. Bush


**Controlled Therapeutic Trials in Cancer. UICC Technical Report Series—Vol 32.** Eds. R. FLAMANT and C. FOHANNO (1978) Geneva: International Union Against Cancer. 684 pp. 28.00 Sw. fr.

This volume is a comprehensive collection, compiled by the UICC, of all clinical trials registered with the UICC information office by June 1977. The plan of data collection is outlined in the introduction to the book, and thereafter a series of tables defines the trial according to the disease site, with further details on the protocol and of the relevant investigating unit. One of the major shortcomings in this volume is the lack of cross-reference by means of an author index but, none the less, for any group of practising clinical research oncologists this is an important summary of currently registered ongoing trials in malignant disease. Although it is unlikely that any individual would want to possess such a volume, it will be an invaluable reference for an oncological library.

H. BUSH